# Nixtamalization of Maize to Reduce Mycotoxin Exposure: A Human Biomonitoring Intervention Study in Soweto, South Africa

**DOI:** 10.3390/toxins17110527

**Published:** 2025-10-26

**Authors:** Elias Maris, Palesa Ndlangamandla, Oluwasola A. Adelusi, Oluwakamisi F. Akinmoladun, Julianah O. Odukoya, Richard T. Fagbohun, Samson A. Oyeyinka, Palesa Sekhejane, Roger Pero-Gascon, Marthe De Boevre, Siska Croubels, Patrick B. Njobeh, Sarah De Saeger

**Affiliations:** 1 Centre of Excellence in Mycotoxicology and Public Health (CEMPH), Faculty of Pharmaceutical Sciences, Ghent University, 9000 Ghent, Belgium; 2 Laboratory of Molecular Bacteriology, Department of Microbiology, Immunology and Transplantation, Rega Institute, KU Leuven, 3000 Leuven, Belgium; 3 Department of Biotechnology and Food Technology, Faculty of Science, Doornfontein Campus, University of Johannesburg, Gauteng 2028, South Africa; 4 Centre of Excellence in Agri-Food Technologies, National Centre for Food Manufacturing, College of Science, University of Lincoln, Holbeach PE12, UK; 5 Faculty of Science, Nelson Mandela Drive Campus, Walter Sisulu University, Mthatha 5099, South Africa; 6 Department of Chemical Engineering and Analytical Chemistry, Institute for Research on Nutrition and Food Safety (INSA-UB), University of Barcelona, 08028 Barcelona, Spain; 7 Laboratory of Pharmacology and Toxicology, Department of Pathobiology, Pharmacology and Zoological Medicine, Faculty of Veterinary Medicine, Ghent University, 9820 Merelbeke, Belgium

**Keywords:** mycotoxin, maize, nixtamalization, Sub-Saharan Africa, human biomonitoring, food safety

## Abstract

Mycotoxin contamination is a global threat to food safety and human health, especially in regions facing food insecurity, such as Sub-Saharan Africa. This intervention study evaluates the effectiveness of nixtamalization, a traditional alkaline cooking method, in reducing mycotoxin levels in maize and corresponding urinary biomarkers of exposure. Forty adult healthy volunteers from an informal settlement in Kliptown, Soweto (South Africa), were randomly assigned to consume control maize or visibly moldy maize subjected to nixtamalization. Nixtamalization achieved a reduction in fumonisin B3 and deoxynivalenol (DON) to unquantifiable or undetectable levels in maize, while reducing fumonisin B1 (FB1), fumonisin B2, and zearalenone (ZEN) by 95%, 95%, and 89%, respectively. Aflatoxin B1 was unquantifiable before and eliminated after treatment. Biomarker analysis revealed that after consumption of either control or nixtamalized maize, urinary levels of FB1, ZEN, and its metabolites α- and β-zearalenol (α- and β-ZEL) did not show significant differences between groups (*p* > 0.05). DON and tenuazonic acid levels were not affected by the intervention (*p* > 0.05), with urinary detection frequencies remaining above 90%. These results demonstrate nixtamalization effectively lowers mycotoxin levels in maize, resulting in exposure levels comparable to control maize, and highlight human biomonitoring as a sensitive tool for evaluating food safety interventions.

## 1. Introduction

Food safety is an integral part of food security and is dependent on producers, food service operators, regulators, and consumers. Guaranteed access to sufficient, nutritious, and safe food that meets the dietary needs of the population requires careful management throughout the food chain, including the handling, preparation, transportation, storage, and distribution of food products [1,2,3]. Improper management of any stage may lead to food loss and waste, economic losses, and increased risk of adverse health effects due to exposure to food contaminants. Global reports on food safety have highlighted growing concerns over such contaminants, identifying them as a major cause of public health problems and socioeconomic burden, particularly in low- and middle-income countries [2]. Among these contaminants, mycotoxins, secondary metabolites produced by filamentous fungi, represent a significant threat to both human and animal health. These toxic compounds can contaminate staple food crops and are associated with various diseases and, in severe cases, mortality. Documented toxicological effects of mycotoxins include immunotoxicity, reproductive toxicity, genotoxicity, and hepato-, renal-, and cardiotoxicity [4,5]. Recent advances in detection technologies, such as chromatographic, immunochemical, and AI-integrated hyperspectral imaging techniques, are enabling faster and more accurate identification of mycotoxin contamination, reinforcing the importance of robust monitoring systems for food safety management [6,7]. Research has reported that the existence of mycotoxins in Africa is posing a threat to food safety and security, as well as the economy of the continent [8,9,10]. Therefore, effective strategies to reduce mycotoxin levels and mitigate their impact are urgently needed. Mycotoxin contamination leads to substantial food losses annually and negatively affects the competitiveness of African agricultural exports from Africa, especially to the European Union, where strict regulations enforce disposal of contaminated products [8,11,12]. Annual expenses in monetary terms on developing and enforcing control of aflatoxins (AFs) alone in Africa may reach 7.5 million USD [12]. A notable initiative is the Partnership for Aflatoxin Control in Africa (PACA), which was established to address aflatoxin contamination through cross-sector collaboration. Despite the efforts of many African countries to align regulations with international standards, challenges persist, including limited access to advanced testing infrastructure, shortage of trained personnel, and funding constraints essential for supporting research [13]. The lack of data required to develop evidence-based policies further hinders the establishment and enforcement of mycotoxin limits. Furthermore, stringent food safety regulations may inadvertently exacerbate food insecurity among economically disadvantaged populations who may resort to consuming contaminated foods out of necessity. On top of that, traditional food habits and informal food markets complicate the enforcement of food safety regulations [13]. These challenges emphasize the urgent need for investments in food safety infrastructure, research, and policymaking. Moreover, harmonization of food safety regulations across African nations will facilitate safer intra-regional trade [14]. This led to the establishment of the African Food Safety Agency (AfFSA) in 2025, tasked with coordinating and standardizing food safety policies across the continent.

Maize (*Zea mays L.*), a multipurpose staple crop widely used for both food and feed, is projected to become the most widely grown and traded crop globally in the next decade, despite its high susceptibility to fungal contamination [15]. Fungal genera such as *Aspergillus, Penicillium, Fusarium,* along with their mycotoxin by-products, have been reported in maize and maize-based products [9]. A study in South Africa characterized a total of 723 fungal isolates from 21 genera and 99 species across 350 maize samples [9]. Mycotoxin incidence in South Africa is not limited to maize but has also been reported in milk, peanuts, apple juice, wheat, barley, and others [8]. Multiple factors promote fungal growth and mycotoxin production, placing South African food commodities at high risk [11]. As an example, fumonisin (FB) risk in the northeastern regions of South Africa is elevated due to the hot and humid climate that fosters fungal infestation of crops and mycotoxin production, in contrast to the drier southern regions [11,16,17,18]. In the northwestern districts, fumonisin B1 (FB1) is the most prevalent mycotoxin, with incidence rates reaching 100% in small-scale farms and over 96% in large commercial farms [19,20]. In the South, hot and dry temperatures support the growth of *Aspergillus flavus*, which is the principal producer of AFs in maize [21]. Additionally, climate change poses a serious threat to maize production globally. Factors such as drought, extreme heat waves, improper storage management, and altered precipitation patterns can reduce the phyto-immunity of crop plants, thereby increasing susceptibility to fungal contamination and mycotoxin production [11,22,23]. Maize serves as a primary dietary staple to the South African population, especially among underprivileged communities such as those residing in informal settlements or squatter camps. These low-income areas are characterized by illegal development, inadequate infrastructure, limited access to safe water and sanitation, and overcrowding [24,25]. Consequently, these communities face considerable barriers to food security and receive relatively less education and training on food safety and proper handling practices [26,27].

To reduce mycotoxin exposure in humans and animals through the consumption of food and feed, numerous mycotoxin reduction techniques have been developed including chemical, biological, and physical methods [28,29,30,31]. Among these methods, nixtamalization is recognized as an efficient and cost-effective reduction technique [32,33,34]. Nixtamalization is a traditional maize processing method originating from the Mesoamerica indigenous people from around 3.000 years ago [35,36]. This process involves cooking and soaking maize in an alkaline solution, usually limewater (Ca(OH)_2_), which improves its nutritional value, flavor, aroma, color, shelf life, microbial activity, and cooking properties [37,38,39,40]. Nixtamalization enhances the bioavailability of nutrients and facilitates the removal of the pericarp (outer skin), making the maize easier to grind and digest [36,39]. In addition to increasing calcium content, nixtamalization enhances the bioaccessibility of nutrients including fiber and niacin, which contribute gut health, prevention of osteoporosis, and reduce the risk of diseases such as pellagra among consumers [37,38,39,40]. Unlike many other chemical methods, nixtamalization does not negatively impact organoleptic qualities or nutritional content and leaves no harmful residues in the processed food [31]. It has been proven that nixtamalization reduces mycotoxin levels in artificially contaminated maize and sorghum [32,33]. The reduction occurs by combination of toxin degradation, alkaline hydrolysis, and physical removal of the pericarp along with mycotoxins contained therein [33].

To better understand the internal exposure to mycotoxins resulting from dietary intake, human biomonitoring allows for the direct assessment of absorbed and metabolized mycotoxins, offering insights that go beyond food contamination data alone. By measuring specific biomarkers of exposure in biological fluids such as urine, it is possible to assess internal exposure and obtain a more accurate reflection of an individual’s exposure, including from particular foods consumed. Incorporating biomonitoring into food safety research bridges the gap between external contamination and actual human health risk. The purpose of this study was to investigate the awareness of residents of Soweto, South Africa about mycotoxin exposure and to evaluate the impact of nixtamalization on naturally infected maize grains with visible fungal growth (moldy maize), as well as on the mycotoxin biomarkers of exposure in resident’s urine after the consumption of nixtamalized maize compared to a control group.

## 2. Results and Discussion

### 2.1. Participant Demographics and Knowledge of Mycotoxins

With the use of questionnaires, demographics and knowledge of mycotoxins among the participants in Kliptown squatter camps in Soweto were investigated (Table 1). The area was selected for this study due to its poor service delivery, low health practices, and lack of prior research on mycotoxin exposure, highlighting the need to address potential risks and raise community awareness [22,24]. This study has a dual purpose of providing nutritious food to families in low socioeconomic areas facing food insecurity and educating them on healthy eating habits, offering both immediate support and affordable long-term tools for a healthier future. Of the 40 participants, the majority were female (67.5%) and between the ages of 18 and 29. Most had completed secondary education (87.5%) and were unemployed (90%). Maize was consumed frequently, with all participants consuming maize at least once per week and 35% reporting daily consumption. Despite maize being a dietary staple, none of the participants were aware of mycotoxins or their health effects. However, 50% reported being aware of or having experienced food contamination caused by fungi. Among those participants, 40% experienced contamination 2–3 times, 45% experienced it 4–6 times, and 10% experienced it more than six times within a six-month period. These findings suggest a lack of awareness regarding mycotoxins, despite frequent exposure to fungal contamination, indicating a critical gap in food safety knowledge among this population. Population characteristics are summarized in Table 1.

Educational background has correlated positively with knowledge or perception of food contamination by fungi and mycotoxins [41]. The findings reported in this study revealed only a minority (12.5%) had a tertiary education certificate. However, none could ascertain the existence of mycotoxins and associated toxic effects. These results show the need for targeted educational interventions that focus on improving food safety knowledge and practices, particularly in communities with limited resources and lower educational backgrounds. Additionally, public health efforts should focus on raising awareness of mycotoxins and encouraging practices that reduce the risk of exposure, such as proper food storage and regular inspection for signs of fungal contamination. This study reveals that although the sampling population has a relatively high maize consumption and half of the participants come into contact with food contaminated with fungi, none of the participants have ever heard about mycotoxins and their health risks. Contamination of mycotoxins in food coupled with low awareness of mycotoxins in a community can cause continuous human exposure. Therefore, more community training needs to be conducted to help manage fungal contamination and exposure to mycotoxins in the community.

### 2.2. Mycotoxin Levels in Maize Porridge

The effect of nixtamalization on mycotoxin concentrations in maize porridge is shown in Table 2, comparing levels detected in the raw moldy maize, nixtamalized maize, and control maize. To avoid processing-related variations, the moldy maize and control maize were cooked in tap water, prepared as a porridge, and dried at 40 °C overnight in parallel with the nixtamalization process, ensuring comparability among the three sample types. In these maize samples, six different mycotoxins were detected: AFB1, DON, FB1, FB2, FB3, and ZEN. FB1, FB2, and ZEN were present in high concentrations (>900 µg/kg) in moldy maize and were substantially reduced after nixtamalization. AFB1 and DON were completely reduced to levels below the limit of detection (LOD), and FB3 to below the lower limit of quantification (LLOQ). Reduction in mycotoxin levels after nixtamalization ranged from 89 to 100% for all mycotoxins within the quantifiable range, exceeding previously described mycotoxin reduction after nixtamalization [32,33]. Notably, FB1, FB2, and ZEN were also present in control maize, though at lower concentrations than in the nixtamalized samples. Results summarized in Table 2 show the effectiveness of nixtamalization in reducing mycotoxin levels in maize samples infected with fungal pathogens. However, no assessment was performed on potential modified mycotoxins that may be formed during the nixtamalization process, nor on the possible degradation products.

In South Africa, most recent regulations on maximum levels of mycotoxins in foodstuffs are set in Section 15 (2024) of the Foodstuffs, Cosmetics, and Disinfectants Act (Act No. 54 of 1972), published in the official Government Gazette [42,43]. Notably, there are currently no established regulatory limits for ZEN in foodstuffs intended for human consumption. For fumonisins (FB1 + FB2) in maize flour, the regulatory threshold is set at 2000 µg/kg [43]. Maize flour samples with initial fumonisin levels of 3332 µg/kg exceeding the regulatory limit were transformed through nixtamalization to a final concentration of 166 µg/kg, well below the allowable limit, highlighting the effectiveness of nixtamalization in reducing mycotoxin contamination below maximum levels. Similarly, the regulatory limits for DON and total aflatoxins in maize flour are set at 1000 µg/kg and 10 µg/kg, respectively [43]. In this study, no mycotoxin levels in the maize porridges that were used for consumption exceeded these established regional regulatory limits.

Other studies investigating the mycotoxin contamination of maize in Transkei (South Africa) found higher concentrations. For example, Shephard et al. (2013) showed contamination levels of FB1, FB2, and FB3 to be almost 10-times higher in both ‘healthy’ (n = 54) and visibly moldy maize (n = 48) [44]. However, the opposite was true for DON and ZEN and no pretreatment such as boiling was carried out [44]. In another study, mean values of FB1 + FB2 contamination in healthy homegrown maize (n = 141) in Transkei was found to be 820 µg/kg or 1142 µg/kg depending on the season, and much lower in Bizana (South Africa) where levels between 140 µg/kg and 542 µg/kg as a mean value were observed [45]. DON contamination in maize occurred at 290 µg/kg (n = 314) in another study in South Africa, which is close to what was observed in the moldy maize from our study [46]. In the same study, ZEN was detected in almost 50% of the samples, but most were below the LOQ [46]. These results highlight the wide variability in mycotoxin concentrations in maize crops, influenced by environmental and geographical factors. Additionally, the presence of modified mycotoxins may further contribute to underestimation of total exposure.

### 2.3. Human Biomonitoring of Multiple Mycotoxins in Urine

**Figure 1:**
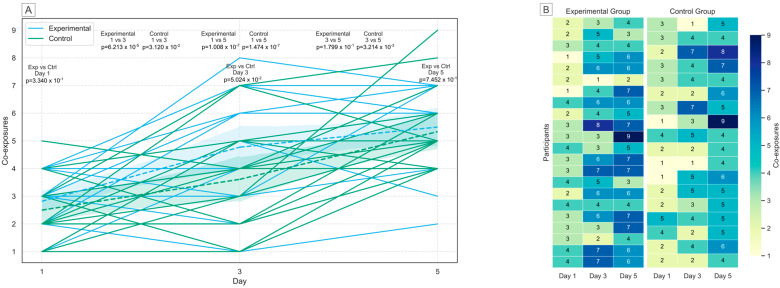
Co-exposures of mycotoxins for all participants over the 5 study days are represented by lines (**A**). *T*-tests with corresponding *p*-values show no significant differences in co-exposures between groups on each day but show a significant difference between study days within each group (except for the experimental group on day 3 vs. day 5). Dotted lines indicates the combined average for all participants in either the experimental (blue) or control (green) group. A day-by-day comparison between the experimental and control groups is provided, with numerical co-exposures reported (**B**).

All 40 participants delivered every urine sample as expected. Figure 1 presents a summary of mycotoxin co-exposures across different days of the study for every participant, stratified by control and experimental group. There was no significant difference in co-exposures between the control and experimental group on any of the sampling days (*t*-test, *p* < 0.05, day 1: *p* = 0.3340; day 3: *p* = 0.0502; day 5: *p* = 0.7452). On the other hand, a significant increase in co-exposures was observed across the different study days (*t*-test, *p* < 0.05). After the consumption of maize on day 5, the amount of co-exposures significantly increased compared to baseline for both groups (control: *p* = 1.474 × 10^−7^; experimental: *p* = 1.008 × 10^−7^), generating a significant increase in the number of mycotoxins participants were exposed to over time. Moreover, no significant increase (*p* = 0.1799) was observed in the experimental group between day 3 and 5 of the study, indicating no further increase in co-exposures after the first consumption period. A summary of these results is presented in Table 3.

The Supplementary Materials (Table S3) provide descriptive statistics for mycotoxins detected in at least one sample, including detection frequency, mean and median concentrations, concentration range, and 95th percentile values, both uncorrected (ng/mL) and creatinine adjusted (µg/g creatinine) for both groups. Mycotoxins detected that are not in detail described in this report include AFB2 (prevalence: 2.5%), AME (2.5%), CIT (45.8%), DOM (8.3), ENN B (0.8%), FUS-X (0.8%), OTA (0.8%), α-ZAL (1.7%), and β-ZAL (15.8%).

**Figure 2:**
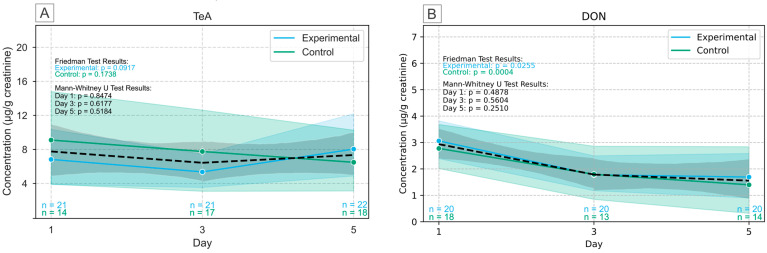
Urinary concentration levels adjusted for creatinine of tenuazonic acid (TeA) (**A**) and deoxynivalenol (DON) (**B**) over the course of the study, indicating no significant difference was observed between groups or between study days. Full lines indicate the average for each group (blue and green). Dotted line (black) indicates the combined average for all participants. The confidence bands (shaded areas) represent the 95% confidence interval.

**Figure 3:**
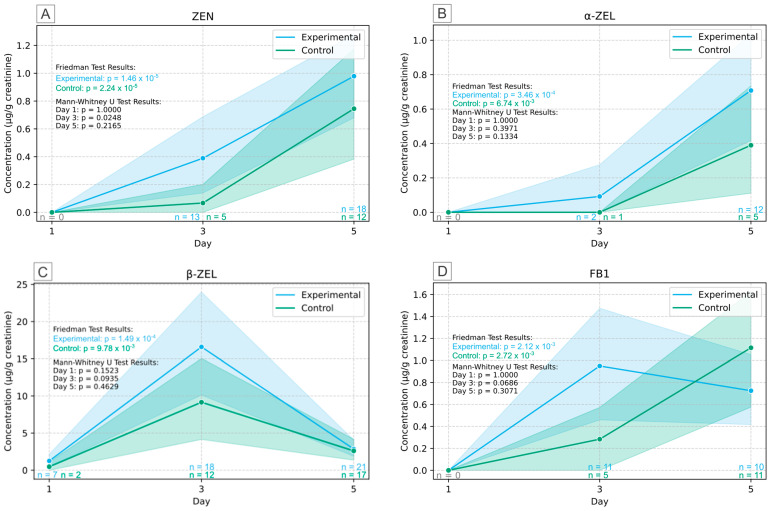
Creatinine adjusted urinary concentration levels of zearalenone (ZEN) (**A**) and its metabolites α-ZEL (**B**) and β-ZEL (**C**) and fumonisin B1 (FB1) (**D**) over the course of the study, visualizing the exposure in control and experimental group and the effect of the intervention after consumption of the prepared maize. Full lines indicate the average for each group (blue and green). The confidence bands (shaded areas) represent the 95% confidence interval.

The results for TeA and DON are shown in Figure 2. TeA and DON had a prevalence of >90% in urine samples but their creatinine-adjusted concentration did not significantly change over the 5-day study period in either the control or experimental group (Friedman, *p* < 0.05). Similarly, on each day of the study, no significant differences were found between the control and experimental group. This underlines that TeA (Figure 3A) and DON (Figure 3B) levels in urine were not affected by the intervention, suggesting that despite the high prevalence in the population, the specific maize samples used did not contribute significantly to a change in internal exposure. Additionally, DON contamination in the moldy maize was low compared to FBs and ZEN. In contrast to DON, TeA was not analyzed in the dried maize porridge samples but is frequently detected in maize, though typically at lower levels than DON [47,48]. It is important to note that the participants did not adhere to a mycotoxin-free diet throughout the course of the study and exposure to these mycotoxins likely originates from sources outside of the maize analyzed in this study.

The results of the human biomonitoring of ZEN and its metabolites are presented in Figure 3. On the first sampling day, urinary levels of ZEN (Figure 3A) were below the LOD. On the third day, the morning after participants consumed two servings of porridge, ZEN levels increased. Furthermore, ZEN measurement on the third day was the only creatinine-adjusted result showing a significant difference between control and experimental group (*p* = 0.0248, n = 11). In contrast, after the second consumption period, ZEN was detected in three times as many participants, and no significant difference between the study groups was observed in day 5 (*p* = 0.2165, n = 30). Alongside ZEN, the metabolite β-ZEL (Figure 3C) peaked after the first consumption on day 3 and dropped to lower levels on day 5. In contrast, α-ZEL (Figure 3B) was increasingly detected on day 5, with frequencies of 28% in the control group and 55% in the experimental group, suggesting a time-dependent metabolization of ZEN. Statistical analysis revealed that β-ZEL and α-ZEL levels were not significantly different in the experimental group compared to the control group during the 5-day study. ZEN and its modified forms are not only formed by metabolization (phase I) in the human body but can also occasionally occur by microbial metabolization in food and feed [49]. Although not common and typically formed under specific post-harvest conditions, α-ZEL, β-ZEL might already be present on the raw maize. However, α-ZEL and β-ZEL were not included in the multiple mycotoxin method used to analyze the dried maize porridges.

Similarly to ZEN, FB1 was not detected on the first day of the study. FB1 was detected on the third (n = 16) and fifth (n = 21) day of the study in the urine of the participants. Also, for FB1, no significant difference was observed in the creatinine-corrected biomonitoring results between the control and the experimental group during the 5-day study (day 1: *p* = 1.000, day 3: *p* = 0.0686, day 5: 0.3071), presented in Figure 3D. The hydrolyzed metabolite HFB1 was not detected in the samples analyzed.

These results show that when exposed to the same mycotoxins at consistent concentrations over a period of multiple days, human biomonitoring levels for some mycotoxins increase over time to detectable levels. However, no significant differences were observed between the experimental and control group three days after the initial consumption. The multiple mycotoxin analysis in maize was reflected in the human biomonitoring results, with the exception of those mycotoxins that were not included in the analysis method for maize (TeA, CIT, ENNB, OTA). The relatively small differences in mycotoxin levels between control maize and nixtamalized maize might also explain the insignificant difference in urinary mycotoxin biomarkers of exposure between control and experimental group. Together, these results emphasize the effectiveness of nixtamalization as a mycotoxin reduction strategy.

When comparing these results with previous studies, it becomes evident that the urinary exposure of DON in this region, which was not detected in the maize consumed in this intervention study, is significantly lower than those reported more than 10 years ago in 2013 [50]. Several factors may contribute to this observation. One important reason is that the natural exposure to this mycotoxin is less, which is a good sign for the public health in this region. Another important consideration is that the analytical method developed and used in this study has much lower limits of detection (LOD) compared to older methods. As a result, average concentrations appear lower, making direct comparisons between studies with different analytical capabilities difficult. This highlights the need to use robust statistics when reporting epidemiological data. While median or percentile values can be compared across studies using methods with differing LODs and/or lower limits of quantification (LLOQ), average values should not be directly compared.

It is hard to make comparisons for mycotoxins that are not measured in the maize consumed during this intervention, as this exposure does not represent population exposure to mycotoxins since an intervention has been done. Furthermore, this is the first ever report for many mycotoxins measured in the urine of an African population.

### 2.4. Implications and Limitations

The findings of this study have important implications for public health, particularly in regions where maize is a staple dietary component. The lack of knowledge combined with the high prevalence of mycotoxin exposure underscores the potential health risks in these communities. There is a need for targeted educational interventions that focus on improving food safety knowledge and practices, particularly in communities with limited resources and a low educational background. Additionally, public health efforts should focus on raising awareness of mycotoxins and encouraging practices that may assist in reducing the risk of exposure. Advocating programs on public awareness of mycotoxin contamination and associated health effects, targeting vulnerable populations particularly in low-income areas, needs to be established countrywide to improve food safety standards. Nixtamalization has been shown to significantly reduce mycotoxin levels in maize through a combination of physical removal and chemical transformation. During the steeping and washing phases, water-soluble mycotoxins such as fumonisins and aflatoxins can be partially leached into the nejayote (cooking liquor), effectively lowering their concentration in the final product. Additionally, the alkaline environment and elevated temperatures used in the process promote chemical degradation or structural modification of mycotoxins into less detectable forms [32,51]. For instance, fumonisins may undergo hydrolysis, resulting in hydrolyzed fumonisin B1 (HFB1), which is considered less toxic than its parent compound but still requires further toxicological evaluation [32]. HFB1 was not included in the method for maize analysis and was not detected in urine. Additionally, the process may also lead to the formation of matrix-bound or modified mycotoxins whose toxicological profiles are not yet fully understood. Despite these reductions, the safety of the resulting degradation or transformation products is not fully understood, and some modified or matrix-bound mycotoxins may persist. The use of liquid chromatography–high-resolution mass spectrometry (LC-HRMS) could be valuable in identifying these products formed during nixtamalization. Therefore, further research is needed to evaluate whether the observed reductions in mycotoxins translate into meaningful reductions in health risk. When this is established, promoting nixtamalization as an affordable food processing technology could help to enhance food safety by reducing mycotoxin levels and their associated risk associated health issues. In addition, the cultural acceptance of nixtamalization as a food practice in South Africa and the global south not only enhances food safety but also improves the nutritional profile of maize by increasing both its handling properties and the bioavailability of key nutrients [36,37,39].

While this study provides valuable insights into the effectiveness of nixtamalization in reducing mycotoxin exposure in a vulnerable population, several limitations must be acknowledged. First, the study design did not include a fully controlled diet, meaning participants continued consuming their usual foods outside of the maize porridge provided, potentially introducing confounding sources of mycotoxin exposure. Second, although nixtamalization significantly reduced detectable levels of several mycotoxins in maize, the study did not assess the formation or toxicity of potential degradation products or modified mycotoxins that may arise during the process. This raises questions about the overall safety of the treated maize and it is therefore important to further study how mycotoxins are degraded during nixtamalization. Third, the relatively small sample size and short duration of the intervention may limit the generalizability and long-term applicability of the findings. Additionally, the absence of a positive control group consisting of participants consuming porridge made from visibly contaminated maize limits the ability to fully quantify the comparative effectiveness of nixtamalization in reducing internal mycotoxin exposure. However, the inclusion of such a group would raise ethical concerns, as intentionally exposing participants to harmful levels of mycotoxins is not permissible.

## 3. Conclusions

This study reveals that the investigated population is not aware of mycotoxins, despite frequently experiencing fungal contamination in their food. This lack of awareness coupled with high dietary mycotoxin levels most certainly contributes to a high risk of mycotoxin exposure, with potentially significant health implications. Providing nutritious food to families that are struggling in low socioeconomic areas and educating them about proper nutrition aims to support their immediate well-being and provides the tools for a healthier future. This is the first report on the effect of maize nixtamalization on internal mycotoxin biomarkers of exposure using a human biomonitoring approach. The findings underscore nixtamalization as an effective method for reducing mycotoxin levels in maize and its related biomarkers of exposure, emphasizing its potential as a food safety intervention. The high prevalence and levels of mycotoxin exposure observed in participant’s urine raises concerns regarding the health of the inhabitants in Soweto when consuming their general diet, mainly consisting of maize and its by-products. Therefore, the need for its conservation may be achieved through good environmental stewardship and personal care by embracing sustainable good agricultural practices to address food safety and sustainability.

## 4. Materials and Methods

### 4.1. Chemicals and Reagents

For urine analysis, mycotoxin standards 3-acetyl-deoxynivalenol (3-ADON), 15-acetyl-deoxynivalenol (15-ADON), aflatoxin B1 (AFB1), aflatoxin B2 (AFB2), aflatoxin G1 (AFG1), aflatoxin M1 (AFM1), beauvericin (BEAU), citrinin (CIT), cyclopiazonic acid (CPA), diacetoxyscirpenol (DAS), deoxynivalenol (DON), deoxynivalenol-3-glucoside (DON-3G), deepoxy-deoxynivalenol (DOM), enniatin A (ENNA), enniatin A1 (ENNA1), enniatin B (ENNB), enniatin B1 (ENNB1), fumonisin B1 (FB1), fumonisin B2 (FB2), fumonisin B3 (FB3), fusarenon-X (FUS-X), fully hydrolyzed-fumonisin B1 (HFB1), neosolaniol (NEO), ochratoxin-alpha (OTα), ochratoxin A (OTA), roquefortin C (ROQ-C), sterigmatocystin (STERIG), T-2 toxin (T-2), zearalanone (ZAN), zearalenone (ZEN), ^13^C_17_-Aflatoxin B_1_ (^13^C_17_-AFB_1_), ^13^C_13_-Citrinin (^13^C_13_-CIT), ^13^C_15_-Deoxynivalenol (^13^C_15_-DON), ^13^C_34_-Fumonisin B_1_ (^13^C_34_-FB_1_), ^13^C_22_-HT-2 toxin (^13^C_22_-HT-2), and ^13^C_24_-T-2 toxin (^13^C_24_-T-2) were purchased from Fermentek (Jerusalem, Israel). Aflatoxin G2 (AFG2), alternariol (AOH), alternariol monomethyl ether (AME), HT-2 toxin (HT-2), nivalenol (NIV), tenuazonic acid (TeA), α-zearalanol (α-ZAL), β-zearalanol (β-ZAL), α-zearalenol (α-ZEL), β-zearalenol (β-ZEL), ^13^C_10_-tenuazonic acid (^13^C_10_-TeA), were purchased from Sigma Aldrich (St. Louis, MO, USA). To correct for sample preparation and ultra-high performance liquid chromatography-tandem mass spectrometry (UHPLC-MS/MS) analysis variability, the ^13^C-labeled standards were fully labelled and used as internal standards when analyzing urine samples.

Standards were combined into a standard mixture with varying concentrations based on their linear range and biological relevance. All ^13^C-labeled internal standards were provided in acetonitrile (ACN) solution or prepared by dissolving the compounds in ACN. The exact concentration of standards was verified using certificates of analysis or adjusted based on calibration with reference standards, ensuring accurate and sensitive quantification. Ultrapure water was obtained from a purified-water system from Sartorius AG (Göttingen, Germany). Sodium chloride (purity 100.0%) and magnesium sulphate (anhydrous, purity 98.6%) were obtained from VWR International (Radnor, PA, USA). Glacial acetic acid (HAc) LC-MS grade (purity 99.95%), formic acid (FA) LC-MS grade (purity 99%), ACN absolute LC-MS grade, and methanol (MeOH) LC-MS grade were purchased from BioSolve (Valkenswaard, The Netherlands). Sodium hydroxide (NaOH) (purity >99%) and ammonium acetate (purity ≥98%) were purchased from Merck (Darmstadt, Germany). Picric acid (1.3% in H_2_O (saturated)) and creatinine powder (MW = 113.12 g/mol) were purchased from Sigma-Aldrich (St. Louis, MO, USA). Calcium hydroxide (Ca(OH)_2_, purity 100%) was obtained from Associated Chemical Enterprises (Johannesburg, South Africa).

### 4.2. Study Population, Design and Ethics Approval

**Figure 4:**
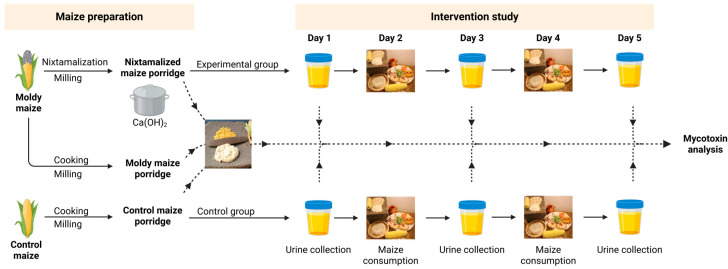
Study design of the intervention trial. Mycotoxin analysis was carried out on three kinds of maize porridges in triplicate. Urine samples were analyzed on day 1, day 3, and day 5 of the study for both experimental (n = 22) and control (n = 18) group. Arrows indicate the maize processing steps and sample collection points throughout the intervention study.

The study population consisted of adult inhabitants of the Kliptown squatter camps located in Soweto, Gauteng, South Africa. A total of forty voluntary participants (n = 40) were recruited for the study through an inclusion health survey questionnaire and consent form. Through questionnaires and face-to-face interviews, information regarding participants’ backgrounds, consumption of maize, and perception of mycotoxin contamination and risks was collected. The study targeted healthy adults aged 18–65 who consumed maize regularly. Participants were educated about the study’s significance, focusing on the role of mycotoxins in food safety and the potential benefits of their involvement. The recruitment process adhered to ethical guidelines approved by the University of Johannesburg Faculty of Science Ethics Committee (ethical clearance number: Odukoya/Ndlangamandla_Njobeh). A written informed consent was obtained for the five-day study, and personal information was encoded to ensure confidentiality. Participants were randomly assigned to the control group (n = 18) or experimental group (n = 22). Equal portions of 300 g maize samples of either control maize or experimental (nixtamalized), prepared as porridge, were consumed both in the morning around 10:30 AM and in the afternoon around 4:00 PM on days 2 and 4. Urine sample collection was carried out before, during, and after the consumption period, on days 1, 3, and 5 respectively, during the early morning (first morning urine). Except for the 4 portions provided on day 2 and day 4, participants adhered to their usual diet, meaning the study did not impose a controlled mycotoxin-free diet. A visual representation of the study design is demonstrated in Figure 4.

Raw maize grains (125 kg of which 12 kg moldy maize) were obtained from two local farmers in Gauteng, South Africa, and stored in silo bags obtained from GLENPAK Pty (Ltd) (Newtown, South Africa). Moldy maize was identified by farmers based on the visible presence of fungal growth. Control maize did not show fungal growth and was considered ready for consumption. The bags were then sent to the University of Johannesburg, where control maize and moldy maize from both farmers were mixed and stored separately at 4 °C for further processing. First morning urine was collected in a transparent container and put on ice. After collecting the urine samples, they were stored at −80 °C at the University of Johannesburg and later shipped frozen to Ghent University, where they were again stored at −80 °C for further multiple mycotoxin analysis.

### 4.3. Processing of Maize and Nixtamalization

Nixtamalization of maize was carried out using a modified method based on Villada et al. (2017) [38], optimized by Odukoya et al. (2021) [33]. The level of contamination in the maize with visible fungal growth, before nixtamalization, was presumed to be very high. It would be unethical to use porridge prepared from the maize grains with visible fungal growth as a (positive) control in the intervention study. To mitigate high contamination risks, moldy maize grains were mixed with control maize grains (7:3 ratio) prior to any treatment or analysis. A total of 70 kg of this mixture of raw maize grains was treated by soaking in alkaline water composed of 140 L of tap water and 700 g of Ca(OH)_2_ (0.5% w/w). The soaked grains were then heated to 92 °C and cooked for 40 min. After cooking, the grains were steeped for 8 h at room temperature, and the nejayote (cooking liquor) was drained off. The nixtamalized maize was rinsed twice with tap water for 60 s and dried overnight at 40 °C. The maize grains were milled to a small particle size (<200 µm) using a universal M20 mill obtained from IKA (Staufen, Germany) and sieved (<1 mm) to produce nixtamalized flour, which was subsequently used to prepare nixtamalized maize porridge. Additionally, both the moldy and control maize grains were cooked in tap water, dried, milled, and prepared as porridge by adding boiling tap water. This process was conducted in parallel to nixtamalization to ensure comparability. To prepare the maize porridges, a total of 36 L of clean tap water was measured into a pot and brought to a boil. Subsequently, 15 kg of maize flour was gradually incorporated while stirring continuously for approximately 5 min to obtain a homogeneous mixture. The resulting slurry was then cooked further under constant stirring for an additional 10 min to ensure thorough cooking [52]. After drying the maize porridges overnight at 40 °C, they were subjected to UHPLC-MS/MS mycotoxin analysis to assess contamination levels in moldy, nixtamalized, and control maize and evaluate the effectiveness of nixtamalization in reducing mycotoxin levels in maize grains [39,53]. Nixtamalized and control maize porridges were consumed by the participants as described in Section 2.1.

### 4.4. Multiple Mycotoxins Analysis of Maize Porridges

Sample preparation of maize and analysis were performed using a modified method based on Odukoya et al. (2021) and Meyer et al. (2019) [33,54]. For mycotoxin analysis, 500 g of both control and moldy maize grains were milled to a small particle size (<200 µm) using a universal M20 mill obtained from IKA (Staufen, Germany). The resulting maize flour was sieved using a 1 mm mesh and thoroughly mixed to ensure homogenization and an optimal mycotoxin extraction. To prepare the porridges, boiling tap water was added to the sieved four. The prepared porridge was dried overnight at 40 °C whereafter mycotoxin analysis was carried out by the SAGL (South African Grain Laboratory NPC, Pretoria, South Africa) according to their in-house validated method based on Meyer et al. [54]. The method is accredited for the analysis of multiple mycotoxins in food and feed samples under ISO 17025 [55]. An extraction solution consisting of MeOH/H_2_O/ACN (25/50/25, *v*/*v*/*v*) was prepared, and 40 mL of this solution was added to 10.00 (±0.05) g of dried maize porridge in triplicate. Thereafter, the sample was blended with an overhead stirrer for 1 min and transported to a 50 mL polypropylene centrifuge tube. Extraction for 15 min was carried out on a mechanical shaker at 260 rpm. Following extraction, the sample was centrifuged at 3000 rpm for 10 min. Then, 5 mL of the supernatant measured into a volumetric flask was diluted with 5 mL of a 25% MeOH in H_2_O (*v*/*v*) solution. The final extract was then filtered through a 13-mm diameter, 0.22-µm pore nylon membrane syringe filter from Membrane Solutions (Shanghai, China) into HPLC amber vials.

The analysis was performed using UHPLC-MS/MS on a Waters Acquity UPLC I-class system (Milford, MA, USA) with an ACQUITY UPLC^®^ BEH-C_18_ column (50 × 2.1 mm, 1.7 μm), operating at 30 °C. The mass spectrometer was a Waters ACQUITY TQD tandem quadrupole system equipped with an electrospray ionization (ESI) source operating in positive ion mode (ESI+), controlled by the MassLynx v4.1 software. The separation utilized a gradient elution with mobile phase A consisting of H_2_O with 0.1% FA and 0.5 mM ammonium acetate and mobile phase B consisting of ACN with 0.1% FA at a flow rate of 0.4 mL/min and a run time of 15 min. Each mycotoxin was analyzed using two product ions in multiple reaction monitoring (MRM), with one product ion selected for quantification and the other for confirmation. Matrix-matched standard solutions containing the 13 mycotoxins of interest (AFB1, AFB2, AFG1, AFG2, DON, 15-ADON, FB1, FB2, FB3, OTA, T-2, HT-2, and ZEN) were routinely prepared for UHPLC-MS/MS calibration. The dried porridge of control maize, moldy maize, and nixtamalized maize samples were measured in triplicate. The method was previously validated in compliance with EC Regulation No. 401/2006 for mycotoxin analysis in foodstuffs and described in detail by Meyer et al. [40,42]. The validation parameters are summarized in Supplementary Materials, Table S1. In contrast to the urine-analysis, no labelled internal standards were used during the mycotoxin analysis in maize. Method performance verification procedures and acceptance criteria outlined in SANCO/12571/2013 were adhered to throughout the analysis [40,43].

### 4.5. Human Biomonitoring of Multiple Mycotoxins in Urine

#### 4.5.1. Instrumentation

Urine sample analysis was performed at Ghent University (CEMPH, MSsmall Expertise Centre) using a Waters Acquity UPLC I-class system (Milford, MA, USA) coupled to a Quattro XEVO TQ-XS mass spectrometer (Waters, Manchester, UK). The instrument parameters were as described in the previous study by Degaldo-Povedano et al., 2025 [56]. Data acquisition and processing were conducted using MassLynx™ version 4.2 and TargetLynx^®^ version 4.2 software (Waters, Manchester, UK). Chromatographic separation was performed on an ACQUITY UPLC^®^ HSS T3 column (2.1 × 100 mm, 1.8 μm) attached to a ACQUITY UPLC^®^ HSS T3 VanGuard (2.1 × 10 mm, 1.8 μm) pre-column, based on Martins et al. [57]. The method had a run time of 18 min and a flow rate of 0.3 mL/min. Gradient elution was performed with mobile phase A (H_2_O/MeOH/HAc; 94/5/1, *v*/*v*/*v*) and mobile phase B (H_2_O/MeOH/HAc; 2/97/1, *v*/*v*/*v*) both containing 5 mM ammonium acetate. The gradient began with 95% mobile phase A, which decreased to 35% A over 7 min. From 7 to 11 min, the percentage of A was reduced further to 25%. Then, mobile phase B increased to 99% by minute 13 and maintained for 1 min. The gradient was then quickly returned to initial conditions (95% A), and the system was allowed to equilibrate until minute 18. The injection volume was 10 µL, and the autosampler temperature was set to 10 °C. Both positive and negative electrospray ionization modes (ESI+ and ESI−) were employed using the ESI source. Similar to the methodology described by Delgado-Povedano et al. (2025), the capillary voltage was set to 3.0 kV for ESI+ and 2.5 kV for ESI−. Source and desolvation temperature were set at 120 °C and 500 °C respectively. Cone gas flow was 150 L/h, desolvation gas flow 800 L/h, collision gas flow 0.15 mL/min, nebulizer 7 bar, ion guide offset 1 at 3.0 and ion guide offset 2 at 0.3 [56]. To ensure accurate identification, two transitions with specific dwell time per analyte were monitored during the MRM.

#### 4.5.2. Urine Sample Pretreatment and UHPLC-MS/MS Method Validation

A QuEChERS-based procedure (Quick, Easy, Cheap, Effective, Rugged, Safe) was used for urine analysis. The sample preparation was optimized from Vidal et al. (2018a) and Martins et al. (2019) to achieve a more efficient and sensitive quantification [57,58]. First, the samples were acclimated at room temperature. A total of 450 µL of the sample was added to a 15 mL Falcon tube. To construct a matrix-matched calibration curve and controls, nine 15 mL Falcon tubes were filled with 450 µL control urine. These control urine samples were obtained from CEMPH (Ghent University) laboratory personnel and pooled. When a mycotoxin was present in these samples, quantification was conducted using a standard addition method. Different volumes of standard mixture were added to 7 control samples to make the calibration curve. A volume of 30 µL of the internal standard mixture and 4 mL of extraction solvent (FA/ACN/H_2_O, 3/52/45, *v*/*v*/*v*) was added to each sample, the 7 samples of the calibration curve, and one of the two remaining control samples. Thereafter, 900 mg of magnesium sulphate and 225 mg of sodium chloride were added to each tube and vortexed for 2 min to complete the extraction process. The samples were then shaken for 30 min on an overhead shaker (Agitelec, Paris, France) and centrifuged for 6 min at 4000 × *g* at room temperature. A 1800 µL aliquot of the supernatant was transferred to a glass tube and dried under a gentle nitrogen stream at 40 °C using a Turbovap LV (Biotage, Uppsala, Sweden). The dry residue was then redissolved in 100 µL injection solvent (mobile phase A/mobile phase B, 60/40, *v*/*v*), vortexed for two minutes, and shortly centrifuged at 4000 rpm. The residue was filtered using a 0.22 μm PVDF Durapore^®^ filter (Millipore, Cork, Ireland) by centrifugation for 5 min at 9000 × *g*. The filtrate was then transferred in a UHPLC vial and ready for UHPLC-MS/MS analysis. The UHPLC-MS/MS method was validated for the determination of 39 mycotoxins and metabolites in urine according to European Commission guidelines (EC No. 2002/657 and EU No. 401/2006) [59,60]. Validation included assessment of limit of detection (LOD), lower limit of quantification (LLOQ), apparent recovery (acceptance range: 80–120%), and precision (acceptance value: <20%) across multiple days. A summary of validation parameters is given in Supplementary Materials, Table S2. Samples exceeding the upper limit of quantification (ULOQ) were diluted and reanalyzed to ensure accurate quantification.

#### 4.5.3. Creatinine-Adjustment in Urine

Quantifying urinary creatinine levels is commonly used for adjusting analyte concentrations for variable dilutions, ensuring biologically relevant and comparable results [57,61]. An in-house spectrophotometric method based on the principle of the Jaffe’s reaction was used for urinary creatinine measurement. First, 3.05 g of NaOH was accurately weighed and dissolved in 600 mL of ultrapure water (NaOH 85 mM). Then, 300 mL of 1.3% picric acid was added. To generate the calibration curve, 100.00 mg creatinine was dissolved in 100.0 mL of ultrapure water. Calibration standards were prepared at concentrations of 0, 2, 5, 10, 20, 50, 100, and 200 µg/mL. In a 96-well plate, 100 µL of each calibration standard was added in duplicate. The samples were diluted 1:20 and 100 µL of each diluted sample was added in triplicate to different wells. To each well, 200 µL of the picric acid reagent was added. The plate was incubated for 15 min on an orbital shaker protected from light. After incubation, the absorbance at 520 nm was measured using a spectrophotometer SpectraMax M5 from Molecular Devices (San Jose, CA, USA). The creatinine quantification method was validated using inter-laboratory reference materials RV72 and RV73 (creatinine in urine) provided by the German external quality assessment scheme (G-EQUAS, Erlangen, Germany). Data analysis was performed using the PRO 7 MAX software.

### 4.6. Statistical Analysis

Descriptive statistics were used to assess the effects of nixtamalization on mycotoxin concentrations and multiple mycotoxin exposures. Descriptive statistics results from human biomonitoring data in urine samples included the prevalence of detection, mean, median, concentration range and 95th percentile for both raw and creatinine adjusted values for each group on day 1, 3, and 5 of the study. Frequencies or means of variables were calculated at group level, being experimental or control. Prevalence of detection (n) was calculated as the number of participants with detectable levels (>LOD) per time point. Co-exposures between the control and experimental groups were assessed at each sampling point using independent *t*-tests to evaluate group-level differences over time. Prior to analysis, concentrations exceeding the 97.5th percentile were removed as outliers. Within-group differences over time (day 1, 3, 5) were assessed using the Friedman test, performed separately for the control and experimental group. This non-parametric test was chosen as it accounts for the repeated-measures design of the study and does not assume normally distributed data. To compare concentrations between groups (experimental vs. control) at each individual time point, Mann–Whitney U tests were used. Statistical results were annotated in the corresponding line plots for clarity. Statistical significance was evaluated at an alpha level of 0.05. Analyses were conducted, and figures were generated in Python (v3.11).

## Figures and Tables

**Table 1:** Demographic, behavioral, and health characteristics, and assessment of the knowledge of multiple mycotoxins of study participants in Kliptown squatter camps in Soweto, South Africa.

Characteristic	Category	Frequency (n)	Percentage (%)
Sex	Male	13	32.5
	Female	27	67.5
Education Background	Secondary	35	87.5
	Tertiary	5	12.5
Race	Black	28	70
	Colored	12	30
Marital status	Married	5	12.5
	Single	35	87.5
Employment Status	Retired	4	10
	Unemployed	36	90
Age Group (Years)	18–27	18	46.2
	27–36	9	23.1
	36–46	7	17.9
	46–55	2	5.1
	55–65	3	7.7
Maize Consumption per Week	1 day/week	11	27.5
	2–3 days/week	6	15
	3–5 days/week	4	10
	5–6 days/week	5	12.5
	Daily	14	35
Suffering from Any Disease or Sickness	No	40	100
Awareness of Mycotoxins	No	40	100
Food Contamination with Fungi	No	20	50
	Yes	20	50
Frequency of Contamination within a 6-month period (if Yes)	Not sure	1	5
	2–3 times	8	40
	4–6 times	9	45
	>6 times	2	10

**Table 2:** Effect of nixtamalization on mycotoxin levels in maize: before and after treatment.

Mycotoxin	Control Maize (µg/kg)	Moldy Maize (µg/kg)	Nixtamalized Maize (µg/kg)	Reduction (%)
AFB1	<LOD	<LLOQ	<LOD	-
DON	<LOD	229	<LOD	100
FB1	69	2336	114	95.1
FB2	<LLOQ	996	52	94.8
FB3	<LOD	340	<LLOQ	100
ZEN	552	7661	843	89.0

Limit of detection (LOD), lower limit of quantification (LLOQ), aflatoxin B1 (AFB1), deoxynivalenol (DON), fumonisin B1 (FB1), fumonisin B2 (FB2), fumonisin B3 (FB3), and zearalenone (ZEN).

**Table 3:** Summary statistics of co-exposures, stratified by group over 5 study days.

Group	Day Comparison	t-Statistic	*p*-Value
Control	Day 1 vs. Day 3	−2.24767	3.120 × 10^−2^
Control	Day 1 vs. Day 5	−6.59378	1.474 × 10^−7^
Control	Day 3 vs. Day 5	−3.17073	3.214 × 10^−3^
Experimental	Day 1 vs. Day 3	−4.44979	6.213 × 10^−5^
Experimental	Day 1 vs. Day 5	−6.41384	1.008 × 10^−7^
Experimental	Day 3 vs. Day 5	−1.36389	1.799 × 10^−1^
Control vs. Experimental	Day 1	0.97857	3.340 × 10^−1^
Control vs. Experimental	Day 3	2.022152	5.024 × 10^−2^
Control vs. Experimental	Day 5	0.327383	7.452 × 10^−1^

## Data Availability

The original contributions presented in this study are included in the article/Supplementary Materials. Further inquiries can be directed to the corresponding author.

## Supplementary Materials

The following supporting information can be downloaded at: https://www.mdpi.com/article/10.3390/toxins17110527/s1, Table S1: Method validation parameters for mycotoxins analysis in maize (µg/kg). Table S2: Method validation parameters for mycotoxins analysis in urine (ng/mL). Table S3: Summary of quantitative uncorrected (ng/mL) and corrected (µg/g creatinine) descriptive statistics for each detected mycotoxin, divided by day and group.

## Abbreviations

The following abbreviations are used in this manuscript:

^13^C	Isotopically labeled carbon
3-ADON	3-acetyl-deoxynivalenol
15-ADON	15-acetyl-deoxynivalenol
ACN	Acetonitrile
AFB1	Aflatoxin B1
AFB2	Aflatoxin B2
AFG1	Aflatoxin G1
AFG2	Aflatoxin G2
AFM1	Aflatoxin M1
AFs	Aflatoxins
AOH	Alternariol
AME	Alternariol monomethyl ether
BEAU	Beauvericin
Ca(OH)_2_	Calcium hydroxide
CEMPH	Centre of Excellence in Mycotoxicology and Public Health
CIT	Citrinin
CPA	Cyclopiazonic acid
DAS	Diacetoxyscirpenol
DOM	Deepoxy-deoxynivalenol
DON	Deoxynivalenol
DON-3G	Deoxynivalenol-3-glucoside
EC	European Commission
ENNA	Enniatin A
ENNA1	Enniatin A1
ENNB	Enniatin B
ENNB1	Enniatin B1
ESI	Electrospray ionization
FA	Formic acid
FB1	Fumonisin B1
FB2	Fumonisin B2
FB3	Fumonisin B3
FUS-X	Fusarenon-X
HFB1	Hydrolyzed fumonisin B1
HAc	Glacial acetic acid
HT-2	HT-2 toxin
INSA-UB	Institute for Research on Nutrition and Food Safety, University of Barcelona
LC-HRMS	Liquid chromatography–high-resolution mass spectrometry
LLOQ	Lower limit of quantification
LOD	Limit of detection
MeOH	Methanol
MRM	Multiple reaction monitoring
NaOH	Sodium hydroxide
NEO	Neosolaniol
NIV	Nivalenol
OTA	Ochratoxin A
OTα	Ochratoxin-alpha
PACA	Partnership for Aflatoxin Control in Africa
ROQ-C	Roquefortin C
SAGL	South African Grain Laboratory
STERIG	Sterigmatocystin
T-2	T-2 toxin
TeA	Tenuazonic acid
UHPLC-MS/MS	Ultra-high performance liquid chromatography–tandem mass spectrometry
ULOQ	Upper limit of quantification
ZAL	Zearalanol
ZAN	Zearalanone
ZEL	Zearalenol
ZEN	Zearalenone
