# Supplementary material for: Nixtamalization of Maize to Reduce Mycotoxin Exposure: A Human Biomonitoring Intervention Study in Soweto, South Africa
Source: Toxins (Basel). 2025 Oct 26;17(11):527. doi: 10.3390/toxins17110527 (PMC12656582; doi:10.3390/toxins17110527)
Supplement: Supplementary file 1 [file toxins-17-00527-s001.zip › toxins-3919252-supplementary.pdf]

Supplementary Materials: Nixtamalization of Maize to Reduce Mycotoxin Exposure: A Human Biomonitoring Intervention Study in Soweto, South Africa

Elias Maris, Palesa Ndlangamandla, Oluwasola A. Adelusi, Oluwakamisi F. Akinmoladun, Julianah O. Odukoya, Richard T. Fagbohun, Samson A. Oyeyinka, Palesa Sekhejane, Roger Pero-Gascon, Marthe De Boevre, Siska Croubels, Patrick B. Njobeh and Sarah De Saeger

Table S1. Method validation parameters for mycotoxins analysis in maize (µg/kg).

Mycotoxin	LOD	LLOQ	ULOQ
AFB1	2.5	5	80
AFB2	2.5	5	80
AFG1	2.5	5	80
AFG2	2.5	5	80
DON	50	100	8000
15-ADON	50	100	8000
FB1	10	20	8000
FB2	10	20	8000
FB3	10	20	8000
OTA	10	20	80
T-2	10	20	8000
HT-2	10	20	8000
ZEN	10	20	8000

Limit of detection (LOD), lower limit of quantification (LLOQ), upper limit of quantification (ULOQ), aflatoxin B1 (AFB1), aflatoxin B2 (AFB2), aflatoxin G1 (AFG1), aflatoxin G2 (AFG2), deoxynivalenol (DON), 15-acetyl-deoxynivalenol (15-ADON), fumonisin B1 (FB1), fumonisin B2 (FB2), fumonisin B3 (FB3), ochratoxin A (OTA), T-2 toxin (T-2), HT-2 toxin (HT-2), and zearalenone (ZEN).

Table S2. Method validation parameters for mycotoxins analysis in urine (ng/mL).

Mycotoxin	LOD	LLOQ	ULOQ
3/15-ADON	0.25	0.46	50.00
AFB1	0.25	0.46	53.00

AFB2	0.012	0.022	4.70
AFG1	0.30	0.55	59.00
AFG2	0.02	0.03	3.45
AME	0.12	0.23	44.00
AOH	0.25	0.46	40.50
BEAU	0.0008	0.0015	0.49
CIT	0.01	0.02	9.55
CPA	0.12	0.22	50.00
DAS	0.0095	0.0174	0.50
DOM	0.15	0.27	50.00
DON	0.11	0.21	44.50
DON-3G	0.12	0.23	48.50
ENNA	0.0011	0.0021	0.50
ENNA1	0.0011	0.0021	1.00
ENNB	0.0011	0.0021	0.49
ENNB1	0.0008	0.0015	0.50
FB1	0.15	0.28	5.95
FB2	0.08	0.15	8.20
FB3	0.22	0.40	12.45
FUS-X	0.73	1.34	63.50
HFB1	0.13	0.23	50.00
HT-2	0.029	0.054	27.00
NEO	0.072	0.132	65.50
NIV	1.30	2.40	52.50
OTA	0.013	0.023	6.30
OTα	0.36	0.67	69.50
ROQ-C	0.28	0.51	100.00
STERIG	0.12	0.22	63.50
T-2	0.0047	0.0087	0.50
TeA	0.78	1.43	50
α-ZAL	0.28	0.55	48.00
β-ZAL	0.42	0.77	52.50

ZAN	0.09	0.16	20.00
α-ZEL	0.27	0.50	44.50
β-ZEL	0.19	0.35	45.50
ZEN	0.06	0.12	77.50

Limit of detection (LOD), lower limit of quantification (LLOQ), upper limit of quantification (ULOQ) 3-acetyl-deoxynivalenol (3-ADON), 15-acetyl-deoxynivalenol (15-ADON), aflatoxin B1 (AFB1), aflatoxin B2 (AFB2), aflatoxin G1 (AFG1), aflatoxin G2 (AFG2), alternariol monomethyl ether (AME), alternariol (AOH), beauvericin (BEAU), citrinin (CIT), cyclopiazonic acid (CPA), diacetoxyscirpenol (DAS), deepoxy-deoxynivalenol (DOM), deoxynivalenol (DON), deoxynivalenol-3-glycoside (DON-3G), enniatin A (ENN A), enniatin A1 (ENNA1), enniatin B (ENNB), enniatin B1 (ENNB1), fumonisin B1 (FB1), fumonisin B2 (FB2), fumonisin B3 (FB3), fusarenon-X (FUS-X), fully hydrolysed-fumonisin B1 (HFB1), HT-2 toxin (HT-2), neosolaniol (NEO), nivalenol (NIV), ochratoxin A (OTA), ochratoxin-alpha (OTα), roquefortin C (ROQ-C), sterigmatocystin (STERIG), T-2 toxin (T-2), tenuazonic acid (TeA), α-zearalanol (α-ZAL), β-zearalanol (β-ZAL), zearalenone (ZAN), α-zearalenol (α-ZEL), β-zearalenol (β-ZEL), and zearalenone (ZEN).

Table S3: Summary of quantitative uncorrected (ng/mL) and corrected (μg/g creatinine) descriptive statistics for each detected mycotoxin, divided by day and group.

Study Group		Parameter	AFB2	AME	CIT	DOM	DON	ENN B	FB1	FUS-X	OTA	TeA	α-ZAL	β-ZAL	α-ZEL	β-ZEL	ZEN
Day 1: Control		Incidence (%)	n.d.	n.d.	27.778	n.d.	100.00	5.56	n.d.	n.d.	5.56	77.78	11.11	11.11	n.d.	11.11	n.d.
	Uncorrected (ng/mL)	Mean	n.d.	n.d.	0.054	n.d.	4.91	0.0005	n.d.	n.d.	0.11	13.77	8.59	5.12	n.d.	9.39	n.d.
		Median	n.d.	n.d.	0.052	n.d.	2.35	0.0005	n.d.	n.d.	0.11	3.79	8.59	5.12	n.d.	9.39	n.d.
		Min	n.d.	n.d.	0.005	n.d.	1.02	0.0005	n.d.	n.d.	0.11	0.39	7.39	1.16	n.d.	4.37	n.d.
		Max	n.d.	n.d.	0.135	n.d.	15.50	0.0005	n.d.	n.d.	0.11	59.62	9.79	9.09	n.d.	14.41	n.d.
		95th Percentile	n.d.	n.d.	0.122	n.d.	15.36	0.0005	n.d.	n.d.	0.11	46.26	9.67	8.70	n.d.	13.90	n.d.
	Corrected for creatinine (µg/g)	Mean	n.d.	n.d.	0.054	n.d.	3.45	0.0002	n.d.	n.d.	0.04	9.10	4.35	3.65	n.d.	4.26	n.d.
		Median	n.d.	n.d.	0.049	n.d.	2.61	0.0002	n.d.	n.d.	0.04	3.52	4.35	3.65	n.d.	4.26	n.d.
		Min	n.d.	n.d.	0.008	n.d.	0.99	0.0002	n.d.	n.d.	0.04	0.28	3.32	1.11	n.d.	3.64	n.d.
		Max	n.d.	n.d.	0.092	n.d.	14.93	0.0002	n.d.	n.d.	0.04	37.64	5.37	6.18	n.d.	4.89	n.d.
		95th Percentile	n.d.	n.d.	0.089	n.d.	8.75	0.0002	n.d.	n.d.	0.04	27.10	5.27	5.93	n.d.	4.83	n.d.
Day 1: Experimental		Incidence (%)	4.55	n.d.	27.273	4.55	90.91	n.d.	n.d.	n.d.	n.d.	95.45	n.d.	18.18	n.d.	31.82	n.d.
	Uncorrected (ng/mL)	Mean	0.30	n.d.	0.028	1.16	5.43	n.d.	n.d.	n.d.	n.d.	25.08	n.d.	6.11	n.d.	7.84	n.d.
		Median	0.30	n.d.	0.024	1.16	3.46	n.d.	n.d.	n.d.	n.d.	5.43	n.d.	3.79	n.d.	5.98	n.d.
		Min	0.30	n.d.	0.010	1.16	1.26	n.d.	n.d.	n.d.	n.d.	0.39	n.d.	2.12	n.d.	1.56	n.d.
		Max	0.30	n.d.	0.059	1.16	18.99	n.d.	n.d.	n.d.	n.d.	326.77	n.d.	14.73	n.d.	20.77	n.d.
		95th Percentile	0.30	n.d.	0.055	1.16	18.75	n.d.	n.d.	n.d.	n.d.	39.51	n.d.	13.11	n.d.	17.25	n.d.
	Corrected for creatinine (µg/g)	Mean	0.28	n.d.	0.025	0.96	3.28	n.d.	n.d.	n.d.	n.d.	20.95	n.d.	3.33	n.d.	3.93	n.d.
		Median	0.28	n.d.	0.020	0.96	2.77	n.d.	n.d.	n.d.	n.d.	5.27	n.d.	3.29	n.d.	3.82	n.d.
		Min	0.28	n.d.	0.008	0.96	0.96	n.d.	n.d.	n.d.	n.d.	0.33	n.d.	1.71	n.d.	1.73	n.d.
		Max	0.28	n.d.	0.055	0.96	7.20	n.d.	n.d.	n.d.	n.d.	303.46	n.d.	5.03	n.d.	7.88	n.d.
		95th Percentile	0.28	n.d.	0.050	0.96	6.44	n.d.	n.d.	n.d.	n.d.	28.08	n.d.	4.80	n.d.	6.95	n.d.
Day 2: control		Incidence (%)	n.d.	n.d.	38.889	11.11	72.22	n.d.	27.78	n.d.	n.d.	94.44	n.d.	16.67	5.56	66.67	27.78
	Uncorrected (ng/mL)	Mean	n.d.	n.d.	0.018	4.37	6.44	n.d.	3.64	n.d.	n.d.	10.61	n.d.	25.91	3.24	26.41	1.86
		Median	n.d.	n.d.	0.020	4.37	1.85	n.d.	3.63	n.d.	n.d.	3.69	n.d.	18.53	3.24	11.51	0.42
		Min	n.d.	n.d.	0.005	3.74	0.21	n.d.	3.37	n.d.	n.d.	0.39	n.d.	14.08	3.24	2.56	0.14
		Max	n.d.	n.d.	0.041	4.99	44.01	n.d.	4.03	n.d.	n.d.	86.28	n.d.	45.11	3.24	103.39	5.39
		95th Percentile	n.d.	n.d.	0.037	4.93	24.79	n.d.	3.96	n.d.	n.d.	49.83	n.d.	42.45	3.24	90.68	4.94
	Corrected for creatinine (µg/g)	Mean	n.d.	n.d.	0.031	1.61	3.61	n.d.	3.47	n.d.	n.d.	7.76	n.d.	10.26	6.25	17.99	2.69
		Median	n.d.	n.d.	0.024	1.61	2.26	n.d.	1.70	n.d.	n.d.	2.12	n.d.	8.64	6.25	9.65	0.76
		Min	n.d.	n.d.	0.004	1.40	0.18	n.d.	1.37	n.d.	n.d.	0.18	n.d.	5.72	6.25	3.12	0.46
		Max	n.d.	n.d.	0.079	1.82	16.44	n.d.	6.49	n.d.	n.d.	31.40	n.d.	16.41	6.25	60.25	10.40
		95th Percentile	n.d.	n.d.	0.073	1.79	10.62	n.d.	6.46	n.d.	n.d.	29.09	n.d.	15.64	6.25	47.80	8.55
Day 2: Experimental		Incidence (%)	9.09	n.d.	36.364	13.64	90.91	n.d.	50.00	n.d.	n.d.	95.45	n.d.	31.82	9.09	81.82	59.09
	Uncorrected (ng/mL)	Mean	0.12	n.d.	0.028	2.21	3.75	n.d.	3.63	n.d.	n.d.	9.67	n.d.	7.19	11.76	57.43	4.41
		Median	0.12	n.d.	0.023	2.29	2.09	n.d.	3.52	n.d.	n.d.	8.69	n.d.	6.33	11.76	38.74	1.58
		Min	0.04	n.d.	0.005	1.54	0.06	n.d.	3.44	n.d.	n.d.	0.39	n.d.	1.68	5.66	4.50	0.68
		Max	0.20	n.d.	0.063	2.80	19.44	n.d.	4.25	n.d.	n.d.	34.36	n.d.	13.22	17.85	310.69	32.41
		95th Percentile	0.19	n.d.	0.061	2.75	12.22	n.d.	4.13	n.d.	n.d.	26.00	n.d.	12.44	17.24	148.01	15.80
	Corrected for creatinine (µg/g)	Mean	0.12	n.d.	0.016	1.42	1.96	n.d.	2.38	n.d.	n.d.	5.36	n.d.	3.16	4.53	29.92	2.10
		Median	0.12	n.d.	0.017	0.92	1.61	n.d.	2.14	n.d.	n.d.	4.03	n.d.	2.97	4.53	22.37	1.26
		Min	0.03	n.d.	0.004	0.53	0.22	n.d.	1.02	n.d.	n.d.	0.19	n.d.	1.25	1.93	3.34	0.19
		Max	0.21	n.d.	0.024	2.82	6.26	n.d.	3.62	n.d.	n.d.	16.12	n.d.	5.29	7.13	124.18	12.95
		95th Percentile	0.20	n.d.	0.024	2.63	4.24	n.d.	3.57	n.d.	n.d.	15.54	n.d.	5.21	6.87	88.90	6.94
Day 3: Control		Incidence (%)	n.d.	11.11	83.333	11.11	77.78	n.d.	55.56	n.d.	n.d.	100.00	n.d.	5.56	27.78	94.44	66.67
	Uncorrected (ng/mL)	Mean	n.d.	0.11	0.032	0.22	4.16	n.d.	4.24	n.d.	n.d.	11.25	n.d.	3.25	3.71	6.19	2.44
		Median	n.d.	0.11	0.028	0.22	0.32	n.d.	3.54	n.d.	n.d.	3.59	n.d.	3.25	3.08	2.77	1.46
		Min	n.d.	0.06	0.010	0.17	0.06	n.d.	2.28	n.d.	n.d.	0.39	n.d.	3.25	0.55	0.20	0.13
		Max	n.d.	0.16	0.063	0.27	28.09	n.d.	11.17	n.d.	n.d.	54.19	n.d.	3.25	7.37	48.97	11.23
		95th Percentile	n.d.	0.16	0.063	0.27	19.37	n.d.	8.51	n.d.	n.d.	36.58	n.d.	3.25	6.83	20.06	7.29
	Corrected for creatinine (µg/g)	Mean	n.d.	0.03	0.028	0.37	1.80	n.d.	2.01	n.d.	n.d.	8.60	n.d.	1.27	1.40	2.76	1.12
		Median	n.d.	0.03	0.022	0.37	0.33	n.d.	1.95	n.d.	n.d.	2.79	n.d.	1.27	1.21	1.67	0.88
		Min	n.d.	0.02	0.007	0.29	0.04	n.d.	0.89	n.d.	n.d.	0.41	n.d.	1.27	0.57	0.42	0.27
		Max	n.d.	0.04	0.095	0.45	12.39	n.d.	3.03	n.d.	n.d.	44.43	n.d.	1.27	2.11	13.27	3.04
		95th Percentile	n.d.	0.04	0.069	0.44	6.99	n.d.	2.98	n.d.	n.d.	30.24	n.d.	1.27	2.09	7.28	2.38
Day 3: Experimental		Incidence (%)	n.d.	4.55	63.636	9.09	77.27	n.d.	50.00	4.55	n.d.	100.00	n.d.	9.09	54.55	95.45	81.82
	Uncorrected (ng/mL)	Mean	n.d.	0.06	0.040	0.79	7.84	n.d.	3.45	1.58	n.d.	18.94	n.d.	8.70	3.80	8.17	3.30

	Median	n.d.	0.06	0.032	0.79	2.71	n.d.	3.36	1.58	n.d.	17.10	n.d.	8.70	3.49	3.48	2.76
	Min	n.d.	0.06	0.010	0.25	0.25	n.d.	2.14	1.58	n.d.	0.02	n.d.	6.94	1.03	0.77	0.43
	Max	n.d.	0.06	0.148	1.33	43.59	n.d.	6.49	1.58	n.d.	51.11	n.d.	10.46	7.24	47.25	10.12
	95th Percentile	n.d.	0.06	0.095	1.28	21.52	n.d.	5.46	1.58	n.d.	50.36	n.d.	10.28	6.42	17.29	9.25
Corrected for creatinine (µg/g)	Mean	n.d.	0.02	0.021	0.55	3.62	n.d.	1.45	0.58	n.d.	9.96	n.d.	3.18	1.53	2.99	1.32
	Median	n.d.	0.02	0.013	0.55	2.28	n.d.	1.30	0.58	n.d.	6.63	n.d.	3.18	1.33	1.97	1.20
	Min	n.d.	0.02	0.006	0.15	0.09	n.d.	0.86	0.58	n.d.	0.01	n.d.	2.52	0.76	0.43	0.40
	Max	n.d.	0.02	0.107	0.96	25.95	n.d.	2.62	0.58	n.d.	50.31	n.d.	3.84	3.49	11.58	3.19
	95th Percentile	n.d.	0.02	0.058	0.92	9.56	n.d.	2.35	0.58	n.d.	35.83	n.d.	3.77	2.61	6.06	2.59

n.d.: not detected, below limit of detection
